# Heerfordt-Waldenström Syndrome With Cardiac Sarcoidosis

**DOI:** 10.7759/cureus.116503

**Published:** 2026-09-18

**Authors:** Camellia Chaudhuri, Salil Ganu, Yoshita Talmale, Sunanda Chaoji, Tanuja Manohar

**Affiliations:** 1 Internal Medicine, N.K.P. Salve Institute of Medical Sciences and Lata Mangeshkar Hospital, Nagpur, IND; 2 Rheumatology, N.K.P. Salve Institute of Medical Sciences and Lata Mangeshkar Hospital, Nagpur, IND; 3 General Medicine, N.K.P. Salve Institute of Medical Sciences and Lata Mangeshkar Hospital, Nagpur, IND; 4 Medicine, N.K.P. Salve Institute of Medical Sciences and Lata Mangeshkar Hospital, Nagpur, IND

**Keywords:** cardiac sarcoidosis, cranial neuropathy, heerfordt-waldenström syndrome, neurosarcoidosis, sarcoidosis

## Abstract

Heerfordt-Waldenström syndrome is a rare presentation of sarcoidosis with only a handful of cases reported worldwide. It consists of a tetrad of uveitis, parotitis, and VII nerve palsy and chronic fever. Symptomatic cardiac involvement in sarcoidosis is an uncommon but potentially lethal manifestation. A timely diagnosis and treatment can result in the prevention of morbidity and mortality. We report a case of a young woman with multisystem sarcoidosis who presented with constitutional features, uveitis, multiple cranial nerve palsies, leptomeningitis, and ankle oligoarthritis. Supportive investigations, including chest imaging, granulomatous lymphadenitis on histopathology, and a negative interferon gamma release assay, helped to confirm the diagnosis. During the course of her hospitalization, she developed symptomatic cardiac conduction block. Her acute symptoms reversed rapidly, and she showed remarkable improvement with aggressive immunosuppression.

## Introduction

Sarcoidosis is a rare multisystem inflammatory granulomatous disease with a prevalence ranging from 2 to 141 per 100,000 population in various regions of the world [1]. Constitutional features, respiratory involvement, non-caseating granulomatous adenopathy, and articular and ophthalmic involvement are the most common systemic features of sarcoidosis [2]. Cardiac and neurosarcoidosis are uncommon, often refractory, and potentially lethal manifestations of sarcoidosis. Cardiac sarcoidosis is responsible for about 50% of all deaths in sarcoidosis, whereas symptomatic arrhythmias are responsible for about 75% of all deaths in cardiac sarcoidosis [3]. Described first by the Danish Ophthalmologist Christian Heerfordt in 1909 as a tetrad of chronic fever, uveitis, bilateral parotid enlargement and cranial nerve palsies (especially facial nerve palsy), the Heerfordt-Waldenström syndrome remains one of the rare manifestations of sarcoidosis, with prevalence ranging between 4.1 and 5.6% among sarcoidosis cases [4]. A case of sarcoidosis in which multisystem involvement with many common, uncommon, and rare manifestations, such as Heerfordt-Waldenström syndrome, is present still remains of interest. The evolution of cardiac sarcoidosis in a case of Heerfordt-Waldenström syndrome has been described in only a handful of case reports.

## Case presentation

A 24-year-old woman was referred to us for the evaluation of a suspected tubercular lymphadenopathy. She had a history of 12 kg weight loss over the last three months, low-grade fever, and progressively enlarging bilateral lymphadenopathy. Apart from these obvious complaints, she also mentioned certain peculiar recent developments. These included slurred speech, difficulty forming a food bolus, diplopia, and headache that improves with eye closure, along with difficulty climbing down stairs.

She had no history of breathlessness, cough, or hemoptysis. She also had not experienced any weakness or paresthesia in the limbs. There was no history suggestive of vascular events, confusion, abnormal behavior, or convulsions in the past. She did not have any history of tuberculosis or contact with a tuberculosis patient in the past. She was up to date with her childhood vaccinations, including Bacillus Calmette-Guérin (BCG) for tuberculosis.

On examination, she was found to have pallor and multiple discrete, nontender, mobile lymph nodes in the bilateral cervical, submandibular, and axillary areas. She had bilateral corneal opacities with no perception of light in the left eye and only perception of light in the right eye. The rest of the neurological examination was suggestive of left oculomotor (III) palsy, right facial (VII) palsy (Figure 1a), and right-sided hypoglossal palsy (Figure 1b). Both her ankles were swollen and tender, suggestive of synovitis. The rest of the systemic examination had no abnormality. With this constellation of insidious-onset constitutional features, generalized lymphadenopathy along with multiple cranial nerve palsies and ocular involvement, we had expanded the scope of our differentials to rule out granulomatosis with polyangiitis (GPA), IgG4-related disease (IgG4RD), sarcoidosis, and lymphomas (Figure 2).

**Figure 1:**
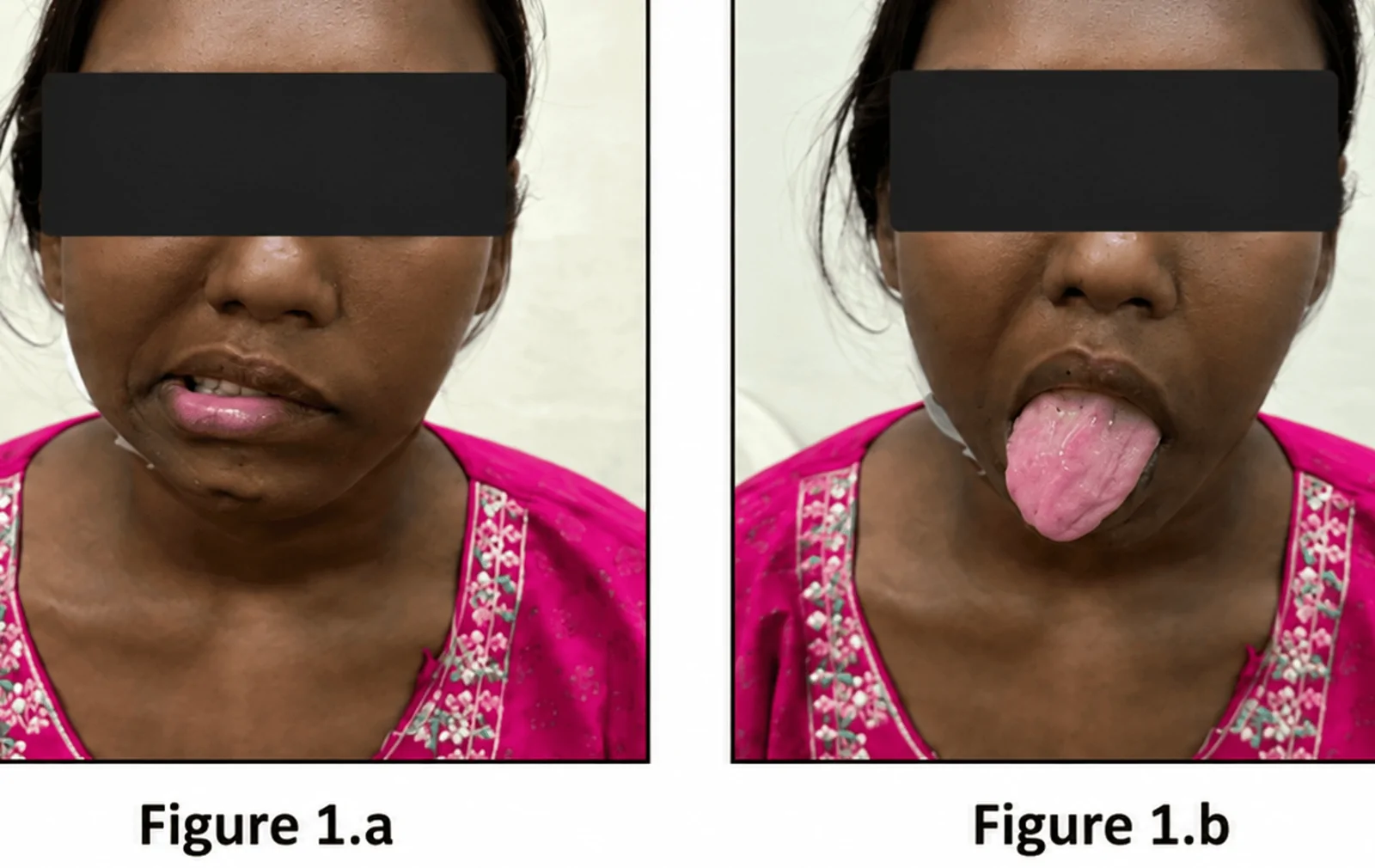
Initial features. (a) VII palsy and (b) XII palsy.

**Figure 2:**
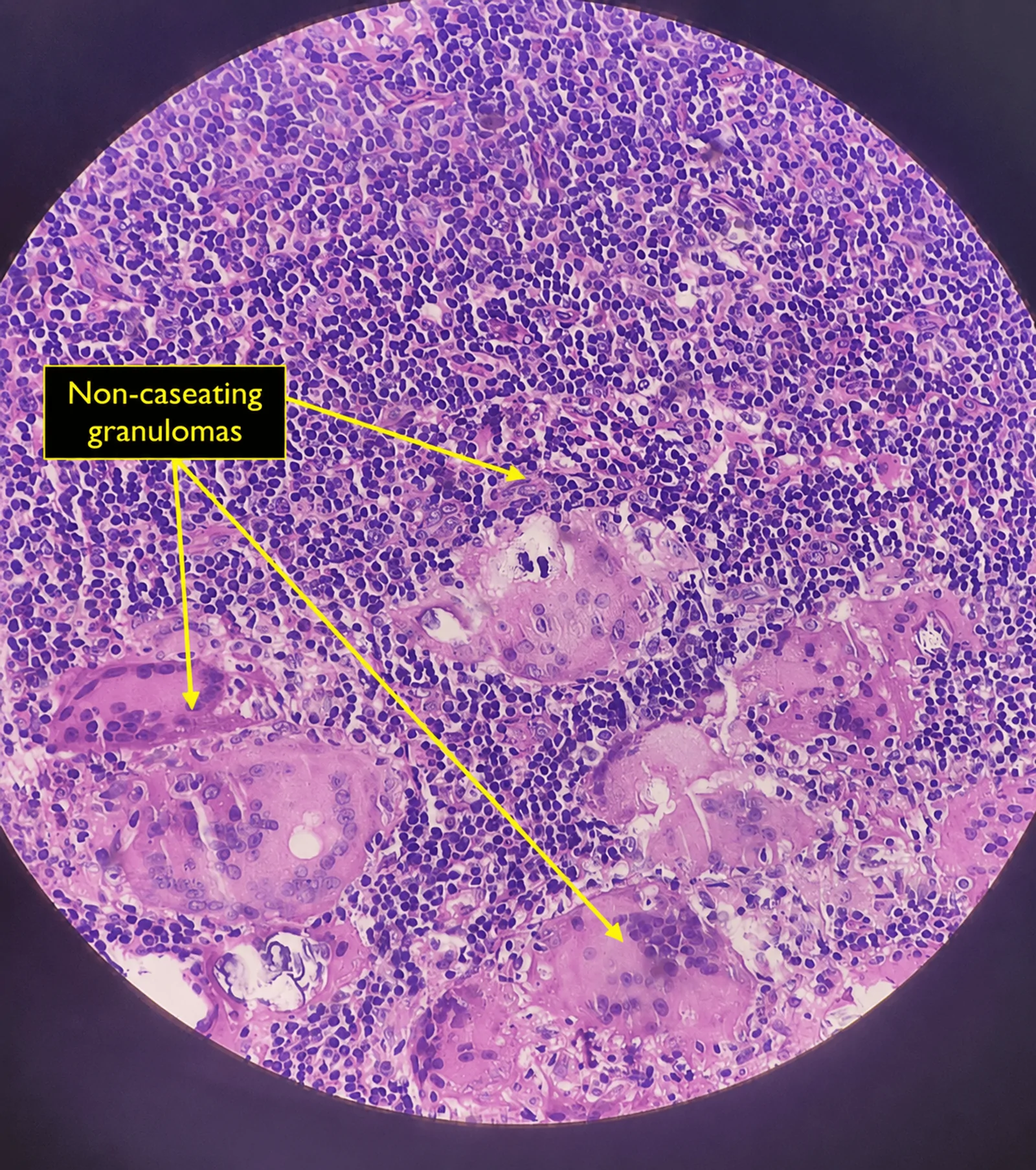
Initial features-cervical lymph node biopsy.

The initial laboratory assessment (Table 1) suggested the presence of microcytic hypochromic anemia (Hb 6.1 gm/dL, mean corpuscular volume (MCV) 67), lymphopenia (total leukocyte count (TLC) 3690/mm^3^ and absolute lymphocyte count (ALC) 990/mm^3^); and raised inflammatory markers (C-reactive protein (CRP) 12 mg/L, erythrocyte sedimentation rate (ESR) 95 mm/h, elevated serum globulin 4.0 g/dl). Guided investigations for the above differentials showed (Table 1) mild hypercalcemia (corrected calcium 10.9 mg/dL) with normal ECG and elevated serum angiotensin-converting enzyme (ACE) levels (130.6 IU/L). Mediastinal widening with normal parenchyma was noted on chest X-ray, and the subsequent high-resolution chest CT scan was suggestive of multiple subcentimetric lymph nodes (Figure 3a). Although her Mantoux was positive, the interferon gamma release assay (IGRA) turned out to be negative. IgG4 levels were normal, and anti-PR3 and anti-myeloperoxidase (MPO) were negative.

MRI of the neck, advised in view of multiple cranial neuropathies, did not show any local growth, but multiple enhancing cervical lymph nodes and bulky submandibular glands were visible (Figure 3b). Contrast-enhanced MRI of the brain showed leptomeningeal enhancement. CSF study was normal (Table 1). The lymph node biopsy from the most prominent cervical lymph node was also done, and a molecular diagnostic test for TB (Gene XPERT) was negative. Therefore, in the context of the above-mentioned clinical features, along with supportive laboratory features like lymphopenia, raised inflammatory markers, raised serum ACE levels, hypercalcemia, very high CRP and ESR; and negative IGRA, anti-MPO and PR3, along with normal IgG4 levels and peripheral smear, a diagnosis of multi-systemic sarcoidosis was made.

A detailed direct history revealed that she had developed bilateral parotitis at the age of 15 years. She had started having a painful redness of the right eye along with photophobia 12 years ago. The same complaints were noticed in the left eye about eight years ago. These progressed slowly till date indicating a chronic bilateral uveitis resulting in corneal opacities causing diminution of vision. She also recalled that during the flares of uveitis, there were ‘mumps-like swelling’ over both cheeks indicating probable parotitis. This raised the suspicion of Heerfordt-Waldenström syndrome.

**Table 1:** Initial laboratory reports and specific tests. ADA: adenosine deaminase; AFB: acid fast bacilli; ALP: alkaline phosphatase; ALT: alanine aminotransferase; AST: aspartate aminotransferase; BNP: B-type natriuretic peptide; CRP: C-reactive protein; ESR: erythrocyte sedimentation rate; GGTP: gamma-glutamyl transferase; HCT: hematocrit; HCV: hepatitis C virus; IGRA: interferon gamma release assay; KFT: kidney function test; LFT: liver function test; LJ: Löwenstein-Jensen; MCH: mean corpuscular hemoglobin; MCHC: mean corpuscular hemoglobin concentration; MCV: mean corpuscular volume; MPO: myeloperoxidase; Na: sodium; NAD: no abnormality detected; NT-proBNP: N-terminal pro-B-type natriuretic peptide; Pr.: protein; RDW: red cell distribution width; TIBC: total iron-binding capacity; TLC: total leukocyte count.

Initial laboratory reports				Specific tests	
CBC		LFT		CSF	
Hb	6.1 g/dL	Bil. D	0.1 µmol/L	Proteins	38.9
MCV	67 fL	Bil. I	0.1 µmol/L	Cells	Acellular
MCHC	29.5 g/dL	ALT	19 IU/L	RBCs	01-Feb
MCH	19.8 pg	AST	20 IU/L	WBCs	Acellular
HCT	20.80%	ALP	103 IU/L	Color	Colorless
RDW	16.80%	Total Pr	7.7 g/L	Appearance	Clear
TLC	3630/mm^3^	A	3.7 g/L	Gram stain	Negative
N	58.10%	G	4 g/L	AFB smear	Negative
L	26.00%	GGTP	NA	LJ C/s	No growth
E	1.90%	KFT		LJ C/s	ADA
M	7.50%	Creatinine	1.15 mg/dL	Cytopath.	NAD
B	0.20%	Urea	35 mg/dL	Parallel Glu.	102
Plt	263,000/mm^3^	Na+	137 mEq/L	Gluc.	81
ESR	95 mm/h	K+	4.3 mEq/L	Other tests	
Retic.	0.60%	Ca++	10.9 mg/dL	Anti-PR3	Negative
Miscellaneous				Anti-MPO	Negative
CRP	12 mg/L	Vitamin D	12.8 nmol/L	Sr. IgG4	128 mg/dL
Sr Iron	88.1	HIV	Negative	IGRA	Negative
TIBC	237	HBsAg	Negative	NT-Pro BNP	912 pg/mL
Mantoux	20 mm	Anti-HCV	Negative	Sr ACE	130.6 IU/L

**Figure 3:**
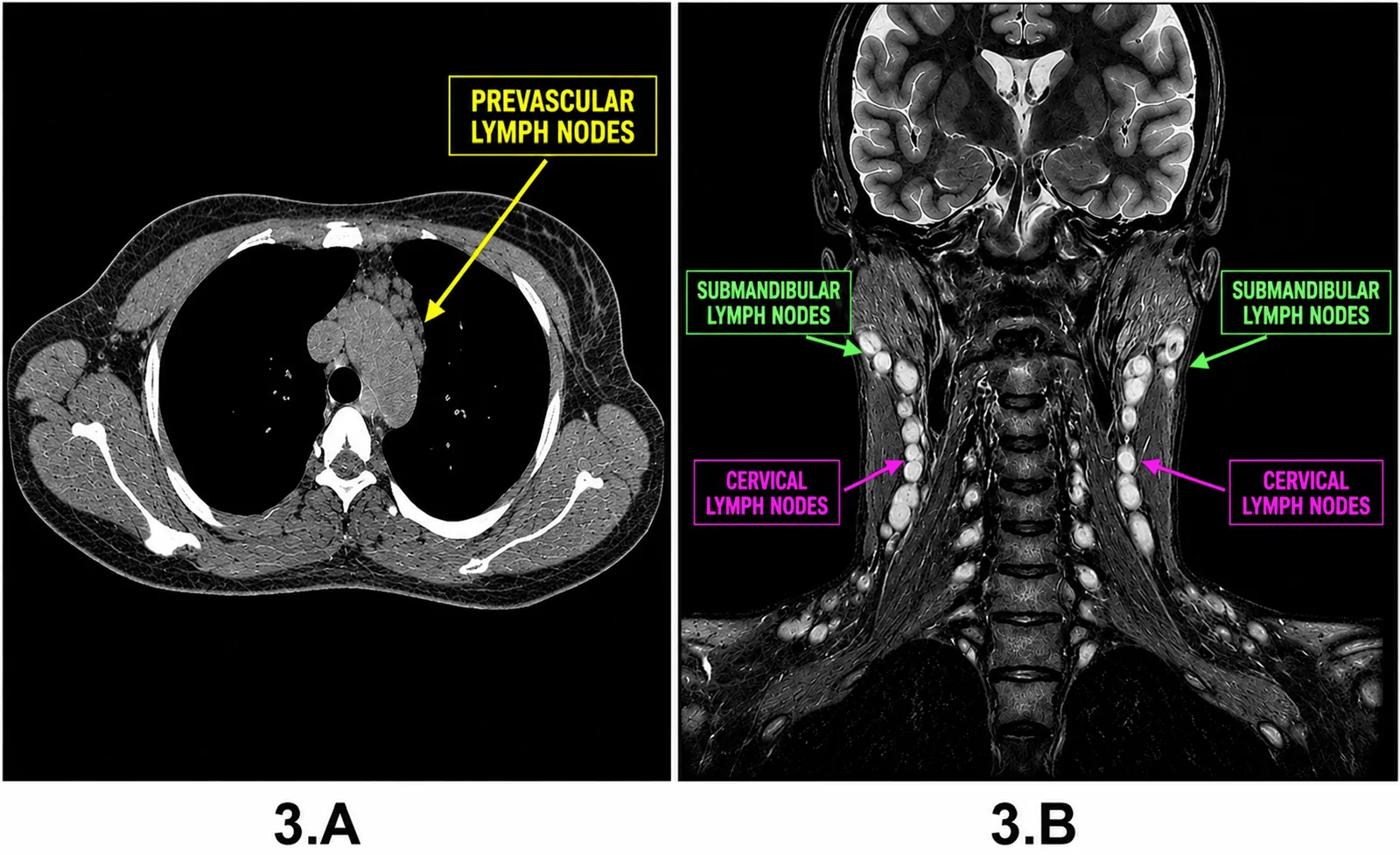
Imaging of (a) HRCT thorax and (b) MRI neck. HRCT: high-resolution computed tomography.

During this workup, she was started on volume expansion with normal saline for asymptomatic hypercalcemia, and her serum calcium level normalized (10.1 mg/dL) by day three. However, by this time we noticed a new bilateral parotid enlargement (Figure 4a). By the next day, she had started getting palpitations. Her ECG showed new PR prolongation (0.28 s) suggestive of a first-degree AV block (Figure 4b). The 2D ECHO showed trivial tricuspid regurgitation but no significant LV wall motion abnormality. Her N-terminal pro-B-type natriuretic peptide (NT-proBNP), however, was elevated (912 pg/mL). A cardiac MRI was deferred due to non-availability. In the above clinical context, the AV block was thought to be attributable to evolving cardiac sarcoidosis. Considering the risk of progression, the patient was started on aggressive treatment with methylprednisolone pulse therapy (1 gm per day for three days) followed by oral prednisolone equivalent to 1 mg/kg/day and subcutaneous methotrexate 15 mg per week. With this treatment, her palpitations stopped, parotid swelling disappeared, and heart block did not worsen. At the time of discharge, her constitutional symptoms had improved; headache, diplopia, and ankle swelling had improved.

**Figure 4:**
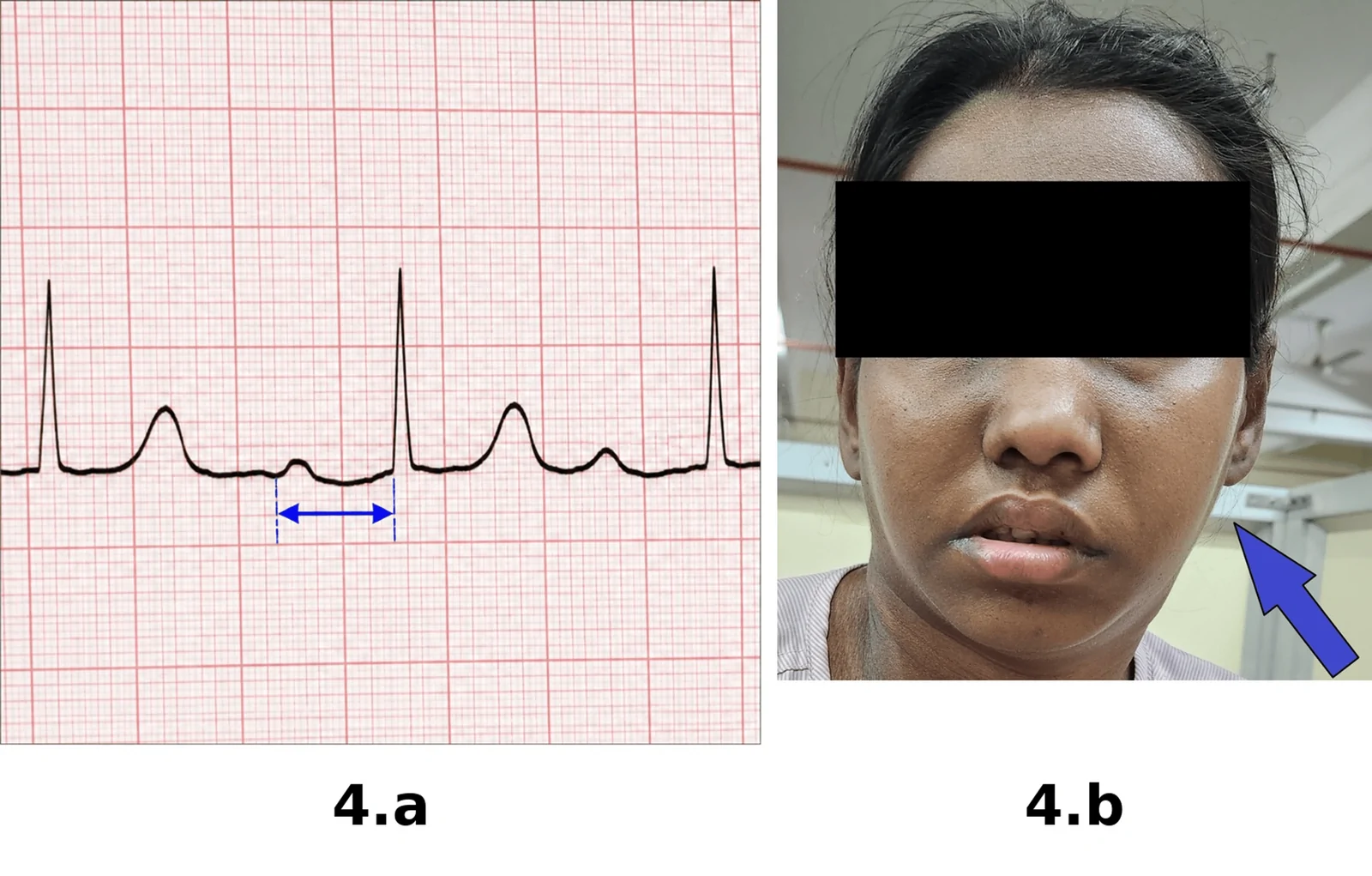
Evolving features. (a) ECG on day three and (b) parotid swelling.

The histopathology report that was available by this time showed a non-caseating granuloma (Figure 2). By her first follow-up, her atrioventricular block had reversed completely, and she had gained 3 kg of weight. Her overall disease timeline is shown in Figure 5.

**Figure 5:**
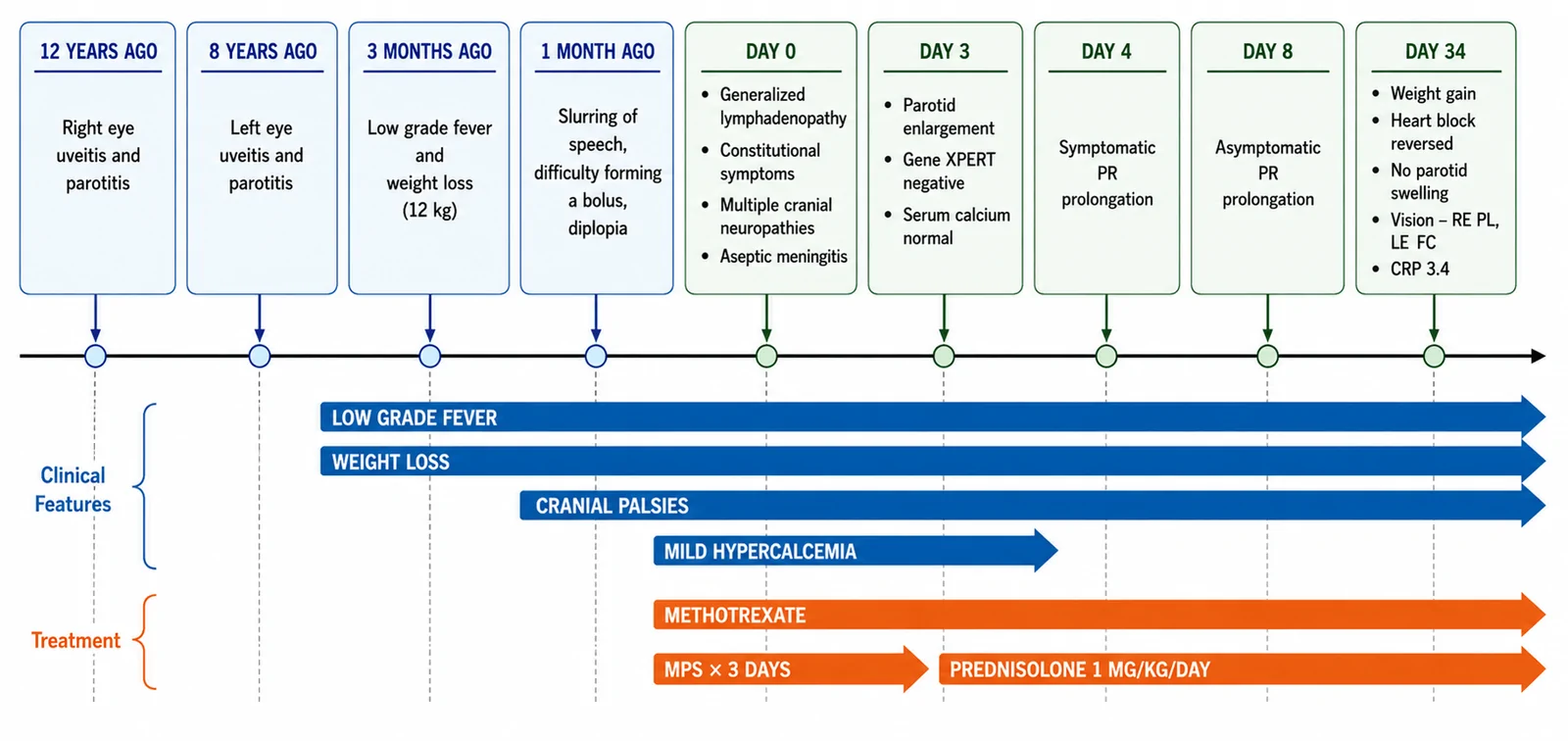
Clinical timeline depicting the evolution of multisystem sarcoidosis, from the onset of ocular and parotid involvement to the development of constitutional, neurological, articular, and cardiac manifestations, followed by initiation of immunosuppressive therapy and subsequent clinical improvement. MPS: methylprednisolone.

## Discussion

The constitutional features, including fever, weight loss, and loss of appetite, have a cumulative prevalence of 33-70% in sarcoidosis [2]. A nontender, generalized granulomatous lymphadenopathy is seen in 15-40% [2]. Early involvement of the ankles is a characteristic feature of sarcoidosis-related arthritis. The overall prevalence of arthritis in sarcoidosis is 10-20% [2]. Although a combination of these features, along with raised inflammatory markers, indicates a systemic granulomatous inflammatory disease, these features are not specific for considering the diagnosis of sarcoidosis. Ocular involvement, typical neurological involvement, hypercalcemia, elevated ACE levels, and evolving cardiac features in this case provided the clues for suspecting the diagnosis. A seemingly trivial history of episodes of “recurrent mumps” starting at the age of 15 years was probably as important. A chronic and nearly silent uveitis was a diagnostic clue that had been overlooked over the years. Its evidence was present in the form of corneal opacities.

The ophthalmic involvement in sarcoidosis is often chronic, asymptomatic, or minimally symptomatic [5]. It can happen at any time during the natural history of the disease, and even if it starts unilaterally, it eventually becomes bilateral over the period of time [6]. Anterior uveitis is the most common form of eye involvement. Uveitis, along with interstitial keratitis, can lead to corneal opacities [5]. The evidence of anterior uveitis in the form of mutton-fat keratic precipitates (KPs) could not be documented through slit lamp due to the presence of corneal opacities. Symptomatic neurological involvement is seen in 5-15% of all cases. Out of these, nearly 50% have facial palsy and about 10% have multiple cranial nerve palsies [7,8]. Involvement of multiple cranial nerves is considered to indicate a severe disease [9]. Granulomatous involvement in sarcoidosis has a predilection for the base of the brain, and leptomeningeal involvement is seen in 20-40% of all cases of neurosarcoidosis [10].

Only 5% of cases of cardiac sarcoidosis are symptomatic, but when present, it often co-exists with neurosarcoidosis and is indicative of a severe disease with high mortality [11]. Sarcoidosis granulomas can directly involve any part of the heart except for the valves. The valvular regurgitation seen in sarcoidosis is often due to papillary muscle infiltration. Conduction abnormalities can range from simple PR prolongation to complete heart blocks and other arrhythmias, including ventricular arrhythmias [12]. Sudden cardiac death is a dreaded complication that prompted the urgent and aggressive treatment in this case. The chronology of systemic involvement with glandular followed by neurological and then cardiac involvement also favored the typical natural history of sarcoidosis. Although this, according to us, was sound logic to start aggressive immunosuppression in anticipation of progression of cardiac blocks to higher degrees, the non-availability of cardiac MRI, PET CT, or a Gallium scan was a major diagnostic hurdle. This was a major limitation for diagnosis of cardiac sarcoidosis as per the consensus criteria provided by the Heart Rhythm Society. This was especially true given that only first-degree heart block (without any other arrhythmia) and abnormal laboratory features were the only cardiac features noted in this case.

While anergy suppressing the hypersensitivity to tuberculin is one of the most commonly accepted explanations for a negative tuberculin skin test (TST) in sarcoidosis, this phenomenon is neither universal nor consistently detected. It is well established that a positive tuberculin test in a case of sarcoidosis is specific for 'tuberculosis infection' (or, by extrapolation, for 'BCG vaccination'), especially in endemic regions [13]. The history of BCG vaccination with a negative IGRA was sufficient to rule out tuberculosis.

The present case had multiple indicators of a severe disease, such as neurological involvement, cardiac sarcoidosis, and Heerfordt-Waldenström syndrome. In addition, the rapid evolution of cardiac features prompted the use of a methylprednisolone pulse followed by high-dose oral steroids, which is the standard of care in these situations [14]. Methotrexate is effective as a first-line agent in pulmonary sarcoidosis [15]. It is also an established treatment option in cardiac sarcoidosis [16].

## Conclusions

Recognizing Heerfordt syndrome provides the crucial diagnostic key that enables prompt multi-modal evaluation, where advanced imaging like cardiac MRI and 18F-fluorodeoxyglucose (FDG) PET is essential to detect potentially fatal cardiac involvement before severe arrhythmias or heart failure manifest. Ultimately, successful management relies on an aggressive, multidisciplinary approach that pairs early systemic immunosuppression with continuous cardiac monitoring to control active granulomatous inflammation, preserve neurological function, and mitigate sudden cardiac death risks.

In a florid multisystem sarcoidosis with multiple poor prognostic factors, including Heerfordt-Waldenström syndrome, neurological involvement, and first-degree conduction blocks, prompt and aggressive immunosuppression averts adverse outcomes.
